# Protocol for characterizing the inhibition of SARS-CoV-2 infection by a protein of interest in cultured cells

**DOI:** 10.1016/j.xpro.2022.101802

**Published:** 2022-10-04

**Authors:** Xinyuan Lai, Hui Zhuang, Tong Li, Kuanhui Xiang

**Affiliations:** 1 Department of Microbiology and Infectious Disease Center, School of Basic Medical Sciences, Peking University Health Science Center, Beijing 100191, China; 2 Peking University-YHLO Joint Laboratory for Molecular Diagnostics of Infectious Diseases, Peking University, Beijing 100191, China

**Keywords:** Cell Biology, Cell-based Assays, Microbiology, Molecular Biology, Protein Bbiochemistry

## Abstract

Here, we present a protocol to characterize the antiviral ability of a protein of interest to SARS-CoV-2 infection in cultured cells, using MUC1 as an example. We use SARS-CoV-2 ΔN trVLP system, which utilizes transcription and replication-competent SARS-CoV-2 virus-like particles lacking nucleocapsid gene. We describe the optimized procedure to analyze protein interference of viral attachment and entry into cells, and qRT-PCR-based quantification of viral infection. The protocol can be applied to characterize more antiviral candidates and clarify their functioning stage.

For complete details on the use and execution of this protocol, please refer to Lai et al. (2022).

## Highlights

• Perform SARS-CoV-2 trVLP system for experiments in biosafety level 2 laboratory
• Detailed steps for experimenting SARS-CoV-2 attachment and entry into cells
• Use MUC1 as an example to assess antiviral function at all infection stages
• Quantify and analyze viral infection by qRT-PCR

## Abstract (editor highlights)

Publisher’s note: Undertaking any experimental protocol requires adherence to local institutional guidelines for laboratory safety and ethics.

## Graphical abstract

**Figure:**
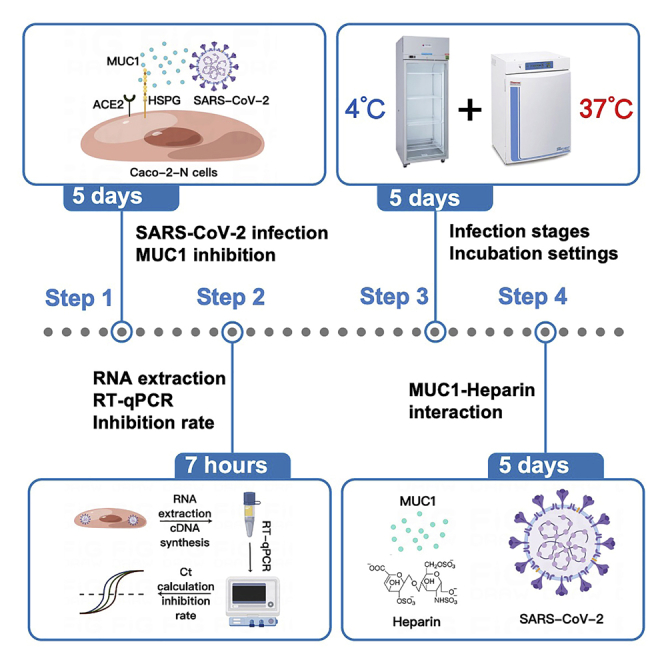

## Teaser

Here, we present a protocol to characterize the antiviral ability of a protein of interest to SARS-CoV-2 infection in cultured cells, using MUC1 as an example. We use the SARS-CoV-2 ΔN trVLP system, which utilizes transcription and replication-competent SARS-CoV-2 virus-like particles lacking nucleocapsid gene. We describe the optimized procedure to analyze protein interference of viral attachment and entry into cells, and qRT-PCR-based quantification of viral infection. The protocol can be applied to characterize more antiviral candidates and clarify their functioning stage.

## Before you begin

The protocol below describes detailed procedures for researchers to investigate the ability of a protein of interest to inhibit SARS-CoV-2 infection in a transcription and replication-competent SARS-CoV-2 virus-like particles lacking nucleocapsid (N) gene (SARS-CoV-2 ΔN trVLP) infection system.

Meanwhile, breast milk inhibits several viral infections, such as human immunodeficiency virus (HIV), cytomegalovirus (CMV), dengue virus, rotavirus, enterovirus, and adenovirus (Kell et al., 2020; van der Strate et al., 2001).

Here we evaluate the antiviral effect of a protein of interest using human breast milk component of mucin1 (MUC1) as an example in a cell infection system of SARS-CoV-2 ΔN trVLP infecting Caco-2-N cells to mimic authentic SARS-CoV-2 infection (Lai et al., 2022; Ju et al., 2021).

***Note:*** Caco-2 are epithelial cells isolated from colon tissue derived from a 72-year-old, white, male patient with colorectal adenocarcinoma (ATCC HTB-37™, https://www.atcc.org/products/htb-37). Caco-2-N cells are established to stably express SARS-CoV-2 N protein by lentiviral transduction. The reason why we choose Caco-2 as infection cell model is that Caco-2 cells express high level of SARS-CoV-2 receptors of ACE2 and TMPRSS2, which are permissive to SARS-CoV-2. Caco-2 cells were widely used to study infection with SARS-CoV and are now again being used to study SARS-CoV2 infections (Bojkova et al., 2020).

***Note:*** SARS-CoV-2 ΔN trVLP expresses a reporter gene (green fluorescent protein, GFP) replacing viral nucleocapsid gene (N), which is required for viral genome packaging and virion assembly. The lack of the viral N protein could be genetically complemented in trans by ectopic expression in Caco-2-N cells. SARS-CoV-2 ΔN trVLP could be propagated and passaged in Caco-2-N cells. If you would like to know more details about the Caco-2-N production, trVLP production and viral titration, you are highly recommended to refer to another published detailed protocol well-written by Prof. Qiang Ding, Tsinghua University (Yu et al., 2021).

The protocol here might also provide clue for other kinds of viruses, such as hepatitis B virus (HBV), hepatitis C virus (HCV), HIV, CMV, dengue virus, Zika virus, etc., to identify the stages of action of their own antiviral candidates by employing different temperature settings.

Before beginning the experiments, we need prepare the protein of interest (commercial MUC1) stock media. According to the product data sheet, the MUC1 commercial product is dissolved in PBS containing 5% Trehalose, pH7.2 to make a 0.1 mg/mL stock.

***Note:*** PBS containing 5% Trehalose is more suitable for dissolving because it can avoid the loss of MUC1 caused by attaching on the tube inner wall.

**CRITICAL:** Centrifuge MUC1 lyophilized protein vial at 10,000 × *g* for 1 min prior to opening. Do not mix by vortex or pipetting. Store at 4°C for 1 week, or store aliquots at −80°C for 3 months. Avoid repeated freezing-thawing.

### Experimental design considerations

The viral infection process can be separated roughly into 3 stages, attachment, entry and post-entry stage, based on the relative position between the virus and cell (Fan et al., 2020; Malone et al., 2022). The attachment stage means that the virus binds to its receptor, which can still happen even at a low temperature of 4°C. The entry stage is a subsequent conformational transition that needs transmembrane serine protease 2 to cleave the spike protein. At 4°C, the enzyme activity is limited so that the conformational transition stops at entry stage. Meanwhile the cell itself uses ATP to achieve cytoskeleton shape change and provides sufficient ATP to overcome the natural barrier between the virus and the cellular membranes to achieve membrane fusion and internalization of viral particles. At 4°C, the ATP enzyme activity is also restricted so that the conformational transition stops. The entry stage happens quickly usually within 1 h at 37°C and costs much energy. The post-entry stage means the virus has been internalized and starts to replicate, which still costs much energy and needs 37°C temperature set (Shang et al., 2020; Fan et al., 2020).

Based on the characteristics of three stages, it works that we use different temperature sets to restrict the viral infection into certain stages. Details of temperature sets are listed below at corresponding section.

A list of all reagents, buffer and equipment for this protocol are described in the key resources table. Make sure all materials required for the experiments are available and/or prepared in advance.

### Institutional permissions

The institution’s Research Ethics Board approval is required when working with human biological material. The human derived samples used in this study was collected with informed consent. The study was approved by the ethics committees of the Medical Center. SARS-CoV-2 is a biosafety level 3 (BSL-3) pathogen. Even though the artificial replicon of trVLP could be used in the BSL-2 laboratory, an appropriate facility and biosafety training for researchers before starting the experiment is needed.

### Cell culture

**Timing: 5–7 days**

1. Thaw a vial of Caco-2-N cells in a 37°C water bath and immediately plate the cells in 10 cm cell culture dishes.

***Note:*** Caco-2-N cells were made from Caco-2 cells transduced with SARS-CoV-2 N gene by lentivirus (pLenti EF1α-SARS-2 N-Flag-BSD), which complements the SARS-CoV-2 GFP/ΔN genome by providing N in trans.

2. Caco-2-N cells (recommend passages: 3–10) are cultured in Basic Dulbecco’s modified Eagle’s media (DMEM) with high glucose and pyruvate and supplemented with 1% Streptomycin/Penicillin and 10% fetal bovine serum (FBS), at 37°C, in 5% CO_2_.
  a. Cells are cultured in 10 cm dishes and split in cell culture media twice per week at a 1:6 ratio. If you need more cells, you can transfer cells to a 175 cm^2^ rectangular angled neck TC-treated culture flask with vent cap (Corning, cat# 431080).
  b. Culture cells for at least 5–7 days after thawing before using them for viral infection to ensure that they are healthy.

**CRITICAL:** All the cells and reagents must be sterile. A strict aseptic environment should be acquired for all the procedures.

## Key resources table

**Table:** 

REAGENT or RESOURCE	SOURCE	IDENTIFIER
**Bacterial and viral strains**		

SARS-CoV-2 ΔN trVLP (GenBank: MN908947)	Established (Ju et al., 2021)	N/A

**Biological samples**		

Human breastmilk	Nanjing Drum Tower Hospital, Nanjing University Medical School, China	N/A

**Chemicals, peptides, and recombinant proteins**		

MUC1	Novoprotein	Cat# CS58
Heparin	HARVEYBIO	Cat# HZB2863-10
Basic Dulbecco’s modified Eagle’s media (DMEM) with high glucose and pyruvate	Gibco	Cat# C11995500BT
TRYPSIN 0.25% EDTA	Thermo Scientific	Cat# 25200072
Streptomycin/Penicillin	BBI	Cat# E607011-0100
Phosphate buffered saline	Invitrogen	Cat# C10010500BT
Fetal bovine serum	Gibco	Cat# 10091148
PBS containing 5% Trehalose, pH7.2	NEOBIOSCIENCE	Cat# NP-1510

**Critical commercial assays**		

FastPure Cell/Tissue Total RNA Isolation Kit	Vazyme	Cat# RC101-01
RevertAid First Strand cDNA Synthesis Kit	Thermo Scientific	Cat# K1622
PowerUp^TM^ SYBR Green Master Mix, 5 ML	Applied Biosystems	Cat# A25742
Cell Counting Kit-8 (CCK-8)	Dojindo	Cat# CK04

**Experimental models: Cell lines**		

Caco-2-N cells	Qiang Ding’s Lab Tsinghua University	N/A

**Oligonucleotides**		

Primer for SARS-CoV-2 RNA (Forward)	Ju et al. (2021)	CGAAAGGTAAGATGGAGAGCC
Primer for SARS-CoV-2 RNA (Reverse)	Ju et al. (2021)	TGTTGACGTGCCTCTGATAAG
Primer for RPS11 (Forward)	This paper	GCCGAGACTATCTGCACTAC
Primer for RPS11 (Reverse)	This paper	ATGTCCAGCCTCAGAACTTC

**Software and algorithms**		

GraphPad Prism version 8	GraphPad Software	v8.0

**Other**		

Cell culture incubator at 37°C, 5% CO_2_	N/A	N/A
Refrigerator at 4°C	N/A	N/A
Precision 180 Water Bath	Thermo Fisher Scientific	Cat# 51221060
100 mm TC-treated Culture Dish	Corning	Cat# 430167
15 mL Centrifuge Tubes	Corning	Cat# 430791
Microcentrifuge Tubes 1.7 mL, sterile	Crystalgen	Cat# 23-2052
0.2 mL PCR Tube	Axygen	Cat# PCR-02-C
48-well Plate	Corning	Cat# 3548
Cell counting slide	Countstar	Cat # CO010101
Centrifuge for 1.5 mL microcentrifuge tubes	N/A	N/A
Applied Biosystems 7500 Fast Real-Time PCR System	qPCR plate reader	N/A
MicroAmpTM Fast Optical 96-Well Reaction Plate with Barcode, 0.1 mL	Thermo Fisher Scientific	Cat# 4346906
MicroAmp™ Optical Adhesive Film	Applied Biosystems	Cat# 4311971

## Materials and equipment

**Table:** Complete DMEM cell culture media

Reagent	Final concentration	Volume (mL)
Basic Dulbecco’s modified Eagle’s media (DMEM) with high glucose and pyruvate	N/A	445
Fetal bovine serum	10%	50
Penicilin / streptomycin	1%	5
**Total**	**N/A**	**500**

***Note:*** Media should be stored at 4°C for no more than 2 months.

## Step-by-step method details

### Caco-2-N cells seeding—Day 1

**Timing: 30 min**

To obtain Caco-2-N cells for SARS-CoV-2 ΔN trVLP infection, cells are firstly collected from the cultured dish.

1. Remove the cell culture media and add 5 mL of sterile PBS to wash the Caco-2-N cells, when Caco-2-N cells grows in a monolayer configuration and at 90% of confluence.
2. Aspirate the PBS and add 2 mL trypsin 0.25% EDTA solution.
3. Incubate at 37°C in the cell culture incubator for 2–5 min.
4. Add 2 mL of complete DMEM media and resuspend the cells.

***Note:*** Pipette the media with cells up-and-down using a 10 mL pipette to better detach and dissociate the cells.

5. Collect the cell suspension in a 15 mL centrifuge tubes.
6. Take an aliquot of 10 μL of cell suspension in a 1.7 mL microcentrifuge tube to count the cell numbers.
  a. Mix the 10 μL cell suspension aliquot with 10 μL trypan blue solution and transfer the 20 μL mixture into a cell counting slide.
  b. Count live cells manually with a hemocytometer or with an automated cell counter to determine the cell density.
7. Prepare the proper volume of cells at the concentration of 2 × 10^5^ cells/mL with the cell culture media.

***Note:*** For example, in this experiment, we have 5 experiment groups and each group has three replicates so that we have to prepare 15 wells of cells. Considering the possible media loss, we are recommended to prepare total volume of 4.5 mL cells for 18 wells (each well needs 50,000 cells in the 250 μL of media). Therefore, we need 18 × 50,000=9 × 10^5^ cells in total. Then, we need prepare 4.5 mL of cell media with cell concentration of 2 × 10^5^ cells/mL. If the cell density of the stock is 2 × 10^6^ cells/mL, we need prepare and mix 450 μL of the stock and 4,050 μL of culture media.

8. Seed 250 μL of cell suspension solution per well in a new 48-well plate.

***Note:*** The data points should be performed at least in triplicates and it should always include a positive control. As for our lab, we choose 2 mg/mL human breast milk sample (A17) which has been tested to inhibit SARS-CoV-2 infection effectively (Fan et al., 2020). Remdesivir is also recommended. As for this test, we test four different concentrations of MUC1 (0.002, 0.0004, 0.00008 and 0 mg/mL).

9. Incubate cells for 18–24 h at 37°C in a CO_2_ (5%) incubator to allow cells to recover and attach to the plate.

### Viral infection—Day 2

**Timing: 3 h**

At this section, we use SARS-CoV-2 ΔN trVLP to infect Caco-2-N cell. We expect to find different infection ratios by adding different concentrations of a protein of interest (e.g., MUC1).

10. Prepare the SARS-CoV-2 ΔN trVLP infection media by adding viral stocks into complete DMEM cell culture media.

***Note:*** In our lab, the viral stock's TCID_50_ exceeds 10^5^. We use SARS-CoV-2 ΔN trVLP to infect Caco-2-N at MOI=1, which can establish stable viral infection in 2 days. The trVLP titration method is in another published protocol (Yu et al., 2021).

11. Prepare different concentrations of a protein of interest (e.g., MUC1) for treating trVLP as follows.

**Table:** 

MUC1 stock solution (μL)	SARS-CoV-2 ΔN trVLP infection media (μL)	PBS containing 5% Trehalose, pH7.2 (μL)	MUC1 concentration (mg/mL)
20	980	0	0.002
4	980	16	0.0004
0.8	980	19.2	0.00008
0	980	20	0

***Note:*** We still need a positive control and a negative control as mentioned above and we recommend you add different volume of stock solution to replace a series of dilution from previous concentration in this experiment actually.

12. Aspirate the cell culture media and add 250 μL of different MUC1-trVLP mixtures respectively into each well as planned.
13. Culture the cells at 37°C, in 5% CO_2_ and incubate for 96 h.

***Note:*** The time is set up depending on the viral replication time and protein stability. The reason why we choose 96 h as our infection endpoint is that we have done time-dependent infection experiment and found 96 h are good enough to extremely expand the inhibitory effects. The cells are going to die and detach from well when extend to 120 h further, which are not suitable to collect cell lysate for RNA extraction.

### Cell collection, RNA extraction, cDNA synthesis, and qRT-PCR detection—Day 6

**Timing: 7 h**

At this section, Caco-2-N cells are harvested for RNA extraction, and then cDNA is synthesized for subsequent qPCR detection.

14. Collect the supernatant and add 250 μL of sterile PBS to wash the cells.
15. Aspirate the PBS and add 250 μL of Buffer RL1 (1% V/V β -mercaptoethanol added) to lyse the cells.
16. Collect the cell lysate into 1.7 mL of RNase-free Microcentrifuge Tubes for RNA extraction.
17. Add Buffer RL2, Buffer RW1, Buffer RW2 and 100 μL of RNase-free ddH_2_O to extract, purify and obtain RNA according to the manufacturer’s instruction (https://www.vazyme.com/download.html?companfileCateId=3).

***Alternatives:*** The protocol shown here is for the FastPure Cell/Tissue Total RNA Isolation Kit following the manufacturer’s instructions (Vazyme, China). Other RNA isolation kits can be used and the respective manufacturer’s protocol should be followed.

18. First Strand cDNA Synthesis using RevertAid First Strand cDNA Synthesis Kit (Thermo Fisher Scientific, USA).
  a. Add the following reagents into a sterile, nuclease-free 0.2 mL PCR Tube on ice in the indicated order:

**Table:** 

RNA template	11 μL
Random Hexamer primer from Synthesis Kit	1 μL
  b. Mix gently, centrifuge briefly and incubate at 65°C for 5 min. Chill on ice, spin down and place the vial back on ice.
  c. Make a premix in the indicated order and add 8 μL premix into each tube containing respective RNA and primer.

**Table:** 

Reagent	1× (μL)	22× (μL)
5× Reaction Buffer	4	88
RiboLock RNase Inhibitor (20 U/μL)	1	22
10 mM dNTP Mix	2	44
RevertAid M-MuLV RT (200 U/μL)	1	22
Total volume	8	176

    ***Note:*** Prepare 1.2 times of premix volume needed to compensate for the liquid loss during pipetting.
  d. Mix gently and centrifuge briefly.
  e. Incubate for 5 min at 25°C for random hexamer primed synthesis, followed by 60 min at 42°C and terminate the reaction by heating at 70°C for 5 min.
19. Set up and load a 96-well plate using the 2× PowerUp^TM^ SYBR Green Master Mix (Applied Biosystems) according to the manufacturer’s instruction (https://www.thermofisher.cn/cn/zh/home/life-science/pcr/real-time-pcr/real-time-pcr-reagents/sybr-green-real-time-master-mixes/powerup-sybr-green-master-mix.html).
  The qPCR primers for viral RNA are as follows:
  SARS-CoV-2-RNA-F: 5′-CGAAAGG TAAGATGGAGAGCC-3′.
  SARS-CoV-2-RNA-R: 5′-TGTTGACGTGCCTCTGATAAG-3′.
  RPS11-F: 5′-GCCGAGACTATCTGCACTAC-3′.
  RPS11-R: 5′-ATGTCCAGCCTCAGAACTTC-3′.
  RPS11 is used to normalize all the data.
  a. Prepare PCR mixture for each well of the 96-well plate:

**Table:** 

Reagent	Volume (μL)
2× PowerUp^TM^ SYBR Green Master Mix	5
Forward primer, 10 μM	1
Reverse primer, 10 μM	1
cDNA template	3
**In total**	10

    ***Note:*** You can make a premix of 2× PowerUp^TM^ SYBR Green Master Mix, Primer-F and Primer-R. If you do so, you need to prepare the 1.2 times of premix volume needed because of the liquid loss during pipetting.
  b. Mix the components thoroughly, then centrifuge briefly to spin down the liquid and eliminate any air bubbles.
  c. Transfer the appropriate volume of each reaction to each well of an optical plate.
  d. Seal the plate with an MicroAmp™ Optical Adhesive Film, then centrifuge briefly to spin down the contents and eliminate any air bubbles.
20. Set up and run the qRT-PCR instrument.
  a. Place the reaction plate in the qRT-PCR instrument.
  b. Set the thermal cycling conditions using the default PCR thermal cycling conditions specified in the following tables according to the instrument cycling parameters and melting temperatures of the specific primers.

**Table:** 

Steps	Temperature	Times	Cycles
UDG activation	50°C	2 min	1
Dual-Lock ™DNA polymerase	95°C	20 s	1
Denature	95°C	3 s	40
Anneal/extend	60°C	35 s	
Dissociation curve conditions (Default)	95°C	15 s	1
	60°C	20 s	
	95°C	15 s	
	60°C	15 s

**Figure 1:**
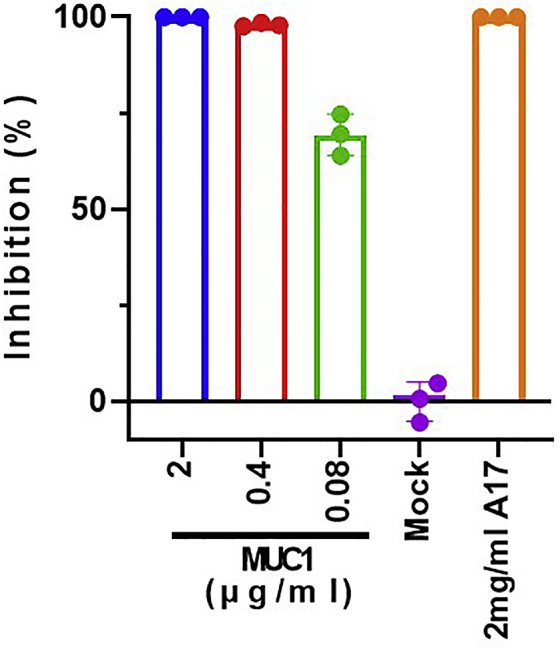
Representative bar graph of an experiment testing the effect of MUC1 on SARS-CoV-2 inhibition Mock means MUC1 is denatured by 100°C for 10 min. Data are represented as mean ± SEM. Data is reproduced from (Lai et al., 2022).

***Note:*** In our lab, we choose 2 mg/mL of human breast milk sample (A17) which has been tested to inhibit SARS-CoV-2 infection effectively (Fan et al., 2020). The inhibition rate test of MUC1 was performed to calculate relative expression compared to the negative control.

21. Analyze data and determine the infection ratio and inhibition effects. MUC1 showed high inhibitory activity to SARS-CoV-2 infection (Figure 1).

### Characterization of a protein of interest in different stages of viral infection process

**Timing: 4–5 days**

The viral infection process can be separated roughly into 3 stages of attachment, entry and post-entry stage. Thus, we use different temperature sets to restrict the viral infection into certain stages. Details of temperature set are listed below at corresponding section.

22. Identification of the antiviral ability of a protein of interest (e.g., MUC1) at attachment stage.
  ***Note:*** The experiment is roughly similar, only the infection process changes. In order to emphasize the differences, the experiments below are focused on the different procedures and simplify the same procedures. The 48 well plate for infection is recommended. The data points should be performed in triplicates.
  a. Prepare three groups of culture media designed as follows:
    i. trVLP infection stock (dilute 10 times).
    ii. trVLP infection stock (dilute 10 times) with 0.002 mg/mL of MUC1.
    iii. trVLP infection stock (dilute 10 times) with 2 mg/mL of A17.
      ***Note:*** Mix these 3 groups respectively and put at 4°C for 1 h.
  b. Add the MUC1-trVLP mixtures mentioned above into the wells for infection and incubate at 4°C for 2 h to allow full viral attachment to cells.
  c. Wash the cells with 300 μL PBS per well for 3 times to remove free MUC1, viral particles and reload 250 μL/well of culture media and culture for 96 h.
  d. Harvest the cells, extract the RNA and carry out qRT-PCR experiment as mentioned above.
    ***Note:*** To better wash away all the free trVLP in the culture media, shake the plates gently after adding the PBS into the wells.
23. Identification of the antiviral ability of a protein of interest (e.g., MUC1) at entry stage.
  At this section, we seed Caco-2-N cells one day prior to viral infection as mentioned above. Caco-2-N cells are kept with trVLP at 4°C for viral attachment, added with MUC1 and carried out to permit viral entry at 37°C. Detailed procedures are listed below:
  a. Dilute the trVLP with complete DMEM cell culture media to MOI=1, then add the viral media into the wells.
  b. Put the plates into a refrigerator at 4°C for 2 h to allow viral attachment enough to cells.
  c. Wash the cells with PBS for 3 times to exclude free virus as mentioned above.
  d. Prepare three groups of media designed as follows:
    i. Fresh media only.
    ii. Fresh media with 0.002 mg/mL of MUC1.
    iii. Fresh media with 2 mg/mL of A17.
  e. Add these 3 groups of media respectively into the wells and transfer to an incubator at 37°C for 1 h to allow viral internalization into cells.
  f. Wash the cells with PBS for 3 times to exclude free viral particles, MUC1 or A17 as mentioned above and reload fresh media and culture for 96 h.
  g. Harvest the cells and analyze the antiviral ability by detecting viral mRNA with qRT-PCR as mentioned above.
24. Identification of the antiviral ability of protein of interest (e.g., MUC1) at post-entry stage. At this section, we seed Caco-2-N cells one day prior to viral infection as mentioned above.
  a. Dilute the trVLP with complete culture media to MOI=1, then add it into the wells and transfer into an incubator at 37°C for 2 h to allow trVLPs fully enter cells.
  b. Wash the cells with PBS for 3 times to exclude free virus.
  c. Prepare fresh media designed as follows:
    i. Fresh media only,
    ii. Fresh media with 0.002 mg/mL of MUC1.
    iii. Fresh media with 2 mg/mL of A17.
  d. Add the prepared media above respectively into the wells and incubate at 37°C, in 5% CO_2_ for 96 h.
  e. Harvest the cells, extracted viral RNA and measure by qRT-PCR to calculate the antiviral ability.

### Possibility of a protein of interest (e.g., MUC1) to inhibit SARS-CoV-2 attaching to heparan sulfate proteoglycans at the attachment stage

**Timing: 4–5 days**

It is reported that SARS-CoV infection can be inhibited by targeting heparan sulfate proteoglycans (HSPG) (Lang et al., 2011) and then SARS-CoV-2 infection depends on both cellular heparan sulfate and ACE2(Clausen et al., 2020). To determine its potential involvement of a protein of interest (e.g., MUC1) in the inhibition of SARS-CoV-2 binding to HSPG, we treated the cells with MUC1 combined with different concentrations of heparin (Lai et al., 2022).

***Note:*** Heparin is an analog of HSPG and can also inhibit viral infection by competitively binding to SARS-CoV-2 thereby preventing viral attachment to HSPG.

In this section, we test and identify whether MUC1 inhibits SARS-CoV-2 attaching to HSPG by using the commercial HSPG analog of heparin. We seed Caco-2-N cells one day prior to viral infection as mentioned above.

25. Prepare different concentrations of heparin mixed with trVLP and MUC1 designed as follows:
  a. trVLP stock (dilute 10 times) only.
  b. trVLP stock (dilute 10 times) + MUC1 (0.002 mg/mL).
  c. trVLP stock (dilute 10 times) + heparin (100 U/mL).
  d. trVLP stock (dilute 10 times) + MUC1 (0.002 mg/mL) + heparin (0.1 U/mL).
  e. trVLP stock (dilute 10 times) + MUC1 (0.002 mg/mL) + heparin (1 U/mL).
  f. trVLP stock (dilute 10 times) + MUC1 (0.002 mg/mL) + heparin (10 U/mL).
  g. trVLP stock (dilute 10 times) + MUC1 (0.002 mg/mL) + heparin (100 U/mL).

***Note:*** Make 4 serial dilutions of MUC1-heparin-trVLP infection media according to the dilution scheme in the table below. The heparin stock is 5,000 U/mL.

The MUC1-trVLP is made by adding 100 μL MUC1 stock (0.1 mg/mL) into 4,900 μL trVLP infection media.

**Table:** 

Dilution series	Heparin (μL)	MUC1-trVLP (μL)	Total dilution factor for heparin
Dilution 1	20 μL heparin stock	980	50×
Dilution 2	100 μL Dilution 1	900	500×
Dilution 3	100 μL Dilution 2	900	5,000×
Dilution 4	100 μL Dilution 3	900	50,000×

26. Add the MUC1-heparin-trVLP mixtures respectively into the wells and incubate at 4°C for 2 h to allow fully viral attachment to cells.
27. Discard the media, wash the cells with 300 μL of PBS per well for 3 times, replenish with fresh media and incubate at 37°C in 5% CO_2_ for 96 h.
28. Collect the supernatant and harvest the cells to extract RNA for further viral RNA quantification by qRT-PCR as mentioned above.

## Expected outcomes

**Figure 2:**
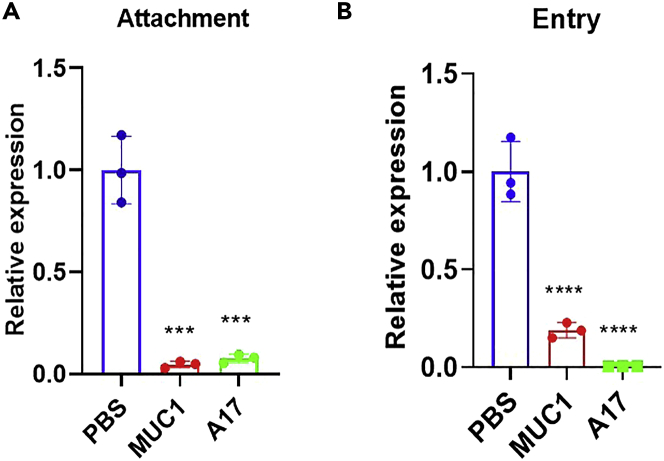
The inhibition of different stages of SARS-CoV-2 infection by MUC1 (A and B) Representative bar graph of an experiment testing the effects of MUC1 on SARS-CoV-2 attaching (A) and entry (B) to the cells. Data are represented as mean ± SEM. Data are reproduced from (Lai et al., 2022).

The protein of interest (MUC1) inhibits SARS-CoV-2 infection and replication in a dose dependent manner (Figure 1). To further determine how these proteins impact viral infection, the exact viral attachment, entry and post-entry experiments were designed to analyze it. It was confirmed that the protein of interest (MUC1) plays a critical role in blocking the steps of viral attachment, entry and post-entry replication (Figure 2).

**Figure 3:**
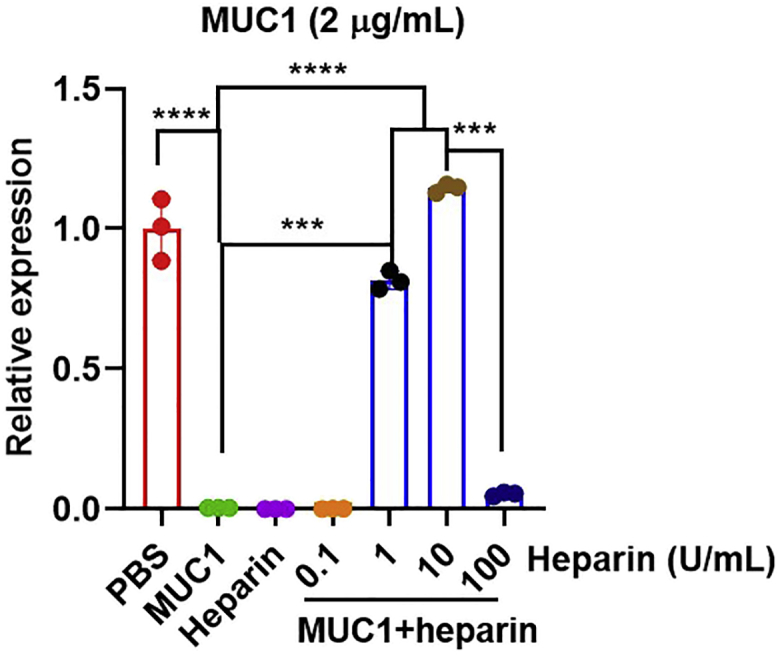
Representative bar graph of an experiment testing the effects of MUC1 and heparin of different combinations on SARS-CoV-2 attaching to the cell inhibition Data are represented as mean ± SEM. Data is reproduced from (Lai et al., 2022).

For the HSPG experiment, we expect that the protein of interest (MUC1) to interfere with the binding of Heparin to SARS-CoV-2. At low concentrations of Heparin, MUC1 is free to bind to HSPG and thereby can block viral attachment. An increase in concentration of Heparin to 10 U/mL increases Heparin-MUC1 binding thereby leaving HSPG free to bind to SARS-CoV-2 making viral attachment possible. Since Heparin can also act as an inhibitor of SARS-CoV-2 binding to HSPG, further increase in Heparin concentration competitively inhibits binding of SARS-CoV-2 to HSPG thereby blocking viral attachment (Figure 3).

## Limitations

The experiment design is based on the SARS-CoV-2 ΔN trVLP and Caco-2-N cells infection system which can mimic the authentic infection in BSL-2 laboratory. But some limitations do exist. First of all, the trVLP system is an artificial replicon which can’t mimic the whole life cycle of SARS-CoV-2. To overcome this limitation, the experiments should be repeated in the live SARS-CoV-2 infection system to prove the proteins interference of viral infection. Moreover, we do not test whether the Caco-2 cells can be replaced by other cell lines (Vero E6 and Huh7.5 expressing SARS-CoV-2 N). We also do find during the passage, SARS-CoV-2 N will decrease its expression probably due to some immunological reasons (The better passages are 5–10 for the experiments). In addition, the construction of trVLP is laborious but the SARS-CoV-2 variants are emerging quickly, various kinds of trVLPs should be developed especially SARS-CoV-2 spike protein replaced by reporter genes (Ricardo-Lax et al., 2021).

When it comes to the protocol details, there are still some limitations. Firstly, we use qRT-PCR to test the MUC1 inhibition ratio, which are likely to be false-positive due to its high sensitivity. Moreover, different kinds of cell lines tolerate temperature shift differently, which will surely influence the MUC1 inhibition efficiency.

## Troubleshooting

### Problem 1

Low viral infection signal in Caco-2-N cells (step 28).

### Potential solution

Check the trVLP titration. In addition, Caco-2-N cells may express low N protein. Add 5 μg/mL of Blasticidin to screen N protein expressing cells.

### Problem 2

Minimal or unexpected changes in MUC1 treatment (step 11).

### Potential solution

The major cause of variation might be improper in MUC1 stock preparation. Uneven cell numbers may also result in unexpected changes. Therefore, it is important to make sure the proper MUC1 stock preparation according to the reconstitution sheet is made. And the same number of cells is seeded in each well of replicates.

### Problem 3

Step 23: very little detected signal exists during the experiment.

### Potential solution

The reason might be the incubation time is not enough for viral attaching to the cells. To solve this, different time points should be tested to help us select the best time point for the experiment.

### Problem 4

Step 24: unexpected result exists in the repeated experiments.

### Potential solution

The virus attaching ability to the cells in the 4°C treatment step is not very tight, which is easily washed away during the wash step. To solve this, it is better to shake the cells gently by the shaker with same time during the wash step.

## Resource availability

### Lead contact

Further information and requests for resources and reagents should be directed to and will be fulfilled by the lead contact, Kuanhui Xiang (kxiang@bjmu.edu.cn).

### Materials availability

This study did not generate new unique reagents.

## Data Availability

This study did not generate or analyze datasets or code.
